# Evolutionary History and Strength of Selection Determine the Rate of Antibiotic Resistance Adaptation

**DOI:** 10.1093/molbev/msac185

**Published:** 2022-09-05

**Authors:** Sandra Cisneros-Mayoral, Lucía Graña-Miraglia, Deyanira Pérez-Morales, Rafael Peña-Miller, Ayari Fuentes-Hernández

**Affiliations:** Programa de Biología Sintética, Centro de Ciencias Genómicas, Universidad Nacional Autónoma de México, 62210 Cuernavaca, Mexico; Department of Cell & Systems Biology, University of Toronto, Toronto, Ontario, Canada; Programa de Biología de Sistemas, Centro de Ciencias Genómicas, Universidad Nacional Autónoma de México, 62210 Cuernavaca, Mexico; Programa de Biología de Sistemas, Centro de Ciencias Genómicas, Universidad Nacional Autónoma de México, 62210 Cuernavaca, Mexico; Programa de Biología Sintética, Centro de Ciencias Genómicas, Universidad Nacional Autónoma de México, 62210 Cuernavaca, Mexico

**Keywords:** antibiotic resistance, experimental evolution, bacterial genomics, mathematical modeling

## Abstract

Bacterial adaptation to stressful environments often produces evolutionary constraints whereby increases in resistance are associated with reduced fitness in a different environment. The exploitation of this resistance-cost trade-off has been proposed as the basis of rational antimicrobial treatment strategies designed to limit the evolution of drug resistance in bacterial pathogens. Recent theoretical, laboratory, and clinical studies have shown that fluctuating selection can maintain drug efficacy and even restore drug susceptibility, but can also increase the rate of adaptation and promote cross-resistance to other antibiotics. In this paper, we combine mathematical modeling, experimental evolution, and whole-genome sequencing to follow evolutionary trajectories towards β-lactam resistance under fluctuating selective conditions. Our experimental model system consists of eight populations of *Escherichia coli* K12 evolving in parallel to a serial dilution protocol designed to dynamically control the strength of selection for resistance. We implemented adaptive ramps with mild and strong selection, resulting in evolved populations with similar levels of resistance, but with different evolutionary dynamics and diverging genotypic profiles. We found that mutations that emerged under strong selection are unstable in the absence of selection, in contrast to resistance mutations previously selected in the mild selection regime that were stably maintained in drug-free environments and positively selected for when antibiotics were reintroduced. Altogether, our population dynamics model and the phenotypic and genomic analysis of the evolved populations show that the rate of resistance adaptation is contingent upon the strength of selection, but also on evolutionary constraints imposed by prior drug exposures.

## Introduction

Antibiotic resistance in response to the industrialized consumption of antibiotics represents one of the most critical problems in human health (Kåhrström 2013; Davies and Davies 2010). Indeed, the scarcity of new antibiotics under development contrasts with the widespread prevalence of resistant strains to all classes of antibiotics discovered to date (Alanis 2005; Årdal et al. 2020). The increasing frequency of drug-resistant pathogens has forced the issue of how to exploit antibiotics best to preserve their efficacy (Bush et al. 2011; Baym et al. 2016). In general, antimicrobial chemotherapy is based on high-dose, long-course antibiotic treatments, a treatment strategy based on the notion that overdosing with antibiotics is, at worst, a therapeutically neutral choice and, as a result, there is no harm in taking drugs for prolonged periods. However, recent clinical studies have argued that the same clinical outcome can be achieved with a short-course antibiotic therapy without imposing such strong selective pressure in favor of drug-resistance (De Waele and Martin-Loeches 2018; Spellberg and Rice 2019).

Other strategies to reduce the selective pressure on any given antimicrobial substance are to appropriately combine different drugs (Chait et al. 2007; Barbosa et al. 2018; Jahn et al. 2021) or to alternate antibiotics in time (Kollef et al. 1997; Niederman 1997). In principle, sequential treatments can minimize resistance by exploiting an evolutionary trade-off, known as collateral sensitivity, in which evolving resistance to one antibiotic causes hypersensitivity to a different drug (Szybalski and Bryson 1952). This strategy is based on the assumption that, by alternating selective pressures, the physiological cost of maintaining resistance mechanisms would be strong enough to select for loss of the resistance allele and thus restore drug susceptibility (Andersson and Hughes 2010). Indeed, there is mounting theoretical (Pena-Miller et al. 2012b; Aulin et al. 2021), clinical (Imamovic et al. 2018), and laboratory (Imamovic and Sommer 2013; Kim et al. 2014; Fuentes-Hernandez et al. 2015; Pál et al. 2015; Roemhild and Schulenburg 2019; Barbosa et al. 2021; Batra et al. 2021) evidence suggesting that sequential treatments can slow the rate of resistance adaptation compared with single-drug treatments.

Unfortunately, restricting the use of antimicrobial substances, either by reducing overall drug consumption (Sabuncu et al. 2009; De Bus et al. 2016) or with treatment protocols based on prioritizing and restricting certain antibiotics (van Duijn et al. 2018), does not necessarily correlate with a decrease in resistance (Beardmore et al. 2017). Moreover, even if drug efficacy is restored after a period of relaxed selection, it is unclear what the rate of resistance acquisition would be if antibiotics were reintroduced into the environment. Do evolutionary trajectories follow the same path as before? Or are there historical contingencies that modify the rate of adaptation when exposed again to antibiotics?

Experimental evolution has been an essential tool to address these questions and to unravel the interaction between selection, chance, and historical contingency in microbial populations (Weinreich et al. 2006; Jansen et al. 2013; Blount et al. 2018; Roemhild et al. 2018; Santos-Lopez et al. 2021). By combining experimental microbiology with whole-genome sequencing, previous studies have been able to follow evolutionary trajectories towards resistance and studied the accumulation of mutations in different ecological contexts. This approach has been widely used to study the evolutionary dynamics that results from combining antibiotics (Hegreness et al. 2008; Pena-Miller et al. 2013; Barbosa et al. 2018; Jahn et al. 2021) or deploying them sequentially (Imamovic and Sommer 2013; Kim et al. 2014; Fuentes-Hernandez et al. 2015; Pál et al. 2015; Roemhild and Schulenburg 2019; Barbosa et al. 2021; Batra et al. 2021), as well as to study adaptation to temporal (Toprak et al. 2012; Lindsey et al. 2013; Oz et al. 2014; Jahn et al. 2017) and spatial (Baym et al. 2016; Fuentes-Hernández et al. 2019) changes in the environmental drug concentration.

A previous study showed that strains that evolved in drug-free environments for over 50,000 generations were more susceptible to most antibiotics than their ancestor, with most of the change occurring during the first 2,000 generations (Lamrabet et al. 2019). When these strains were challenged to a range of drug concentrations, evolved mutants showed, on average, a reduced capacity to evolve resistance relative to their ancestor, thus suggesting that genetic background influences evolutionary pathways towards phenotypic resistance (Card et al. 2019). In a follow-up study, whole-genome sequencing revealed that resistance was produced by divergent genetic changes from exposure to different drugs, but also a consequence of different genetic backgrounds (Card, Thomas, et al. 2021).

Other studies have shown that, as selection increases, the benefit associated with drug-resistance mutations is enhanced, thus increasing mutant frequency in the population and reducing overall genetic diversity. Consequently, strong selective pressures often display similar phenotypic trajectories towards resistance (Toprak et al. 2012) and are known to mitigate the effect of historical contingencies (Pennings 2012; Santos-Lopez et al. 2021). Population bottlenecks are also major determinants of the repeatability of adaptation as they affect genetic drift (De Visser and Krug 2014). Previous studies have shown that bottleneck sizes are important drivers of the evolutionary dynamics of drug resistance (Martínez 2012; Vogwill et al. 2016; Mahrt et al. 2021), with intermediate bottlenecks associated with a high degree of parallel evolution (Vogwill et al. 2016; Garoff et al. 2020; Mahrt et al. 2021; Windels et al. 2021), and that resistance adaptation is maximized under low antibiotic selection and severe bottlenecks, but also under weak bottlenecks and strong selection (Mahrt et al. 2021).

In this paper, we combine a population dynamics model with experimental evolution and whole-genome sequencing to show that bacterial populations evolved under a severe bottleneck and different selective pressures produce multiple genotypes with similar levels of resistance. We will show that resistance mutations selected under mild selection are stable in the population in the absence of selection and, in consequence, the rate of resistance adaptation increased when antibiotics were reintroduced into the environment. In contrast, strongly selected mutants were cleared from the population once the antibiotic was withdrawn.

## Results

We performed a parallel evolutionary experiment consisting of clonal populations of *Escherichia coli* MG1655 evolving to time-varying concentrations of ampicillin (AMP), a β-lactam antibiotic that is prescribed in the clinic for upper respiratory tract infections. The evolutionary experiment consists of three phases. First, we introduced a clonal population into a gradient of 22 logarithmically spaced antibiotic concentrations (supplementary table S1, Supplementary Material online). After 24 h, we diluted one of these populations 1:50 and transferred a sample into another antibiotic gradient with replenished growth media. We repeated this process daily until all evolved populations exhibited a 10-fold resistance compared to the ancestral strain. We refer to this selective phase of the experiment as Phase 1.

For Phase 2, we transferred a sample of each evolved population into drug-free environments and performed serial dilutions for seven days. Finally, Phase 3 consists of transferring populations sampled from the end of Phase 2 into another adaptive ramp (following the same serial dilution protocol used in Phase 1). During all phases, we determined every day the critical drug concentration such that the optical density was below detectable limits (${\mathrm{OD}}_{600}<0.05$), a critical dose we refer to as the minimum inhibitory concentration (MIC). A population sample was frozen for further phenotypic analysis and genome sequencing every time we detected an increase in MIC (see supplementary fig. S1, Supplementary Material online).

### Rate of Adaptation Correlates with the Intensity of the Selective Pressure

**Fig. 1.**
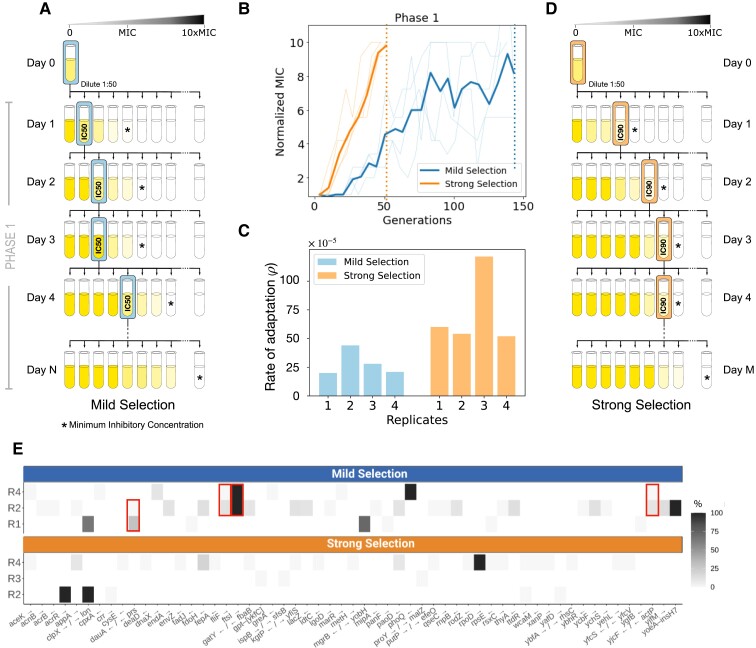
Adaptive ramp with different strengths of selection. (*A*) Schematic illustration of the mild selection regime (MS), consisting of exposing a clonal population (Day 0) to a range of 22 drug concentrations (increasing AMP concentrations are represented from left to right, and MIC is denoted with a star). After 24 h of growth, we measure the resulting optical density achieved in each drug concentration (illustrated in color gradient) and obtain a sample of the population that exhibited 50% inhibition with respect to the drug-free control (Day 1). Thereafter, the selected population (highlighted by the box) is used to inoculate another dose–response experiment (Day 2). We repeat this process until observing an order of magnitude increase in resistance in the evolved populations (Day N). (*B*) Resistance levels (measured in terms of normalized MIC) as a function of time, for both selection regimes (MS in dark, SS in light). Each line denotes an independent replicate, with the mean resistance represented by the solid line. Notably, 10× MIC is achieved in 7 days under SS and 22 days under MS (dotted lines). (*C*) Rate of adaptation estimated for each replicate exposed to adaptive ramps with different strengths of selection (bars represent replicates evolved under MS, and bars under SS). In all replicates, resistance adaptation is accelerated under SS. (*D*) Illustration of an adaptive ramp with strong selection. This protocol is based on transferring daily the population surviving in the highest drug concentration (denoted with a box) into another dose–response experiment. As with MS, the serial transfer experiment was performed until achieving a 10-fold increase in resistance relative to Day 1. (*E*) Mutated genes identified after exposing a clonal population to adaptive ramps with different strengths of selection. Each row represents a different replicate population (MS on top, and SS at bottom). Only mutations not present in the drug-free control, and with frequencies of at least 10% or that appeared in more than one population are shown (top: genes selected for during Phase 1 in MS, bottom: SS. Supplementary tables S2–S3, Supplementary Material online for a complete list of mutations). Squares illustrate genes with mutations in more than one replicate population.

For the selective phases of the experiment (Phase 1 and Phase 3), we implemented adaptive ramps following two different transfer protocols: a mild selection regime (MS; illustrated fig. 1*A*) consisting of transferring daily the population exhibiting 50% inhibition with respect to the drug-free control (IC50), and a strong selection regime (SS; fig. 1*D*), where we transfer each day a sample obtained from the environment with the highest concentration of antibiotics with observable growth (∼IC90). We inoculated four independent replicates for each selection regime from an ancestral clonal population (referred to as WT). Supplementary figure S2, Supplementary Material online illustrates how the concentration of ampicillin the transferred population was exposed to increase as the population became resistant to the antibiotic.

By experimental design, all populations achieved comparable resistance levels at the end of the adaptive ramp, although the number of generations elapsed before reaching the target resistance varied dramatically between selection regimes. In the MS regime, a 10-fold increase in resistance was achieved, on average, after 106.2±38.4 generations, while populations exposed to SS reached the same level of resistance after only 40.8±5.4 generations (fig. 1*B*). This result is consistent with previous studies that have tracked variants in time and reported an accelerated rate of fixation of resistant mutations when evolving under SS than under MS (Mahrt et al. 2021).

To quantify the difference in the adaptation of the populations between the two regimes, we estimated the rate of adaptation in terms of the total resistance improvement achieved in N generations, $\Delta \text{MIC}={\text{MIC}}_{N}-{\text{MIC}}_{0}$, and the interpolated time at which resistance of the population reached half its maximum value, measured in generations, $t_{\mathrm{adapt}}$. Then we quantified the rate of adaptation as $\rho =\Delta \text{MIC}/(2*t_{\mathrm{adapt}})$ (Hegreness et al. 2008; Pena-Miller et al. 2013). As illustrated in figure 1*C*, the rate of adaptation for replicates evolved under SS is larger (mean ρ=0.006, s.e.=0.0001, n=4) than replicates evolved under the mild selection regime (mean ρ=0.002, s.e.=0.0003, n=4; two-tailed t-test, p- value<0.001).

### Genome Sequencing Revealed that Strength of Selection Shaped Mutation Spectra

We performed whole-genome sequencing of the ancestral strain and six replicate populations at the end Phase 1 (three for MS and three for SS). First, note that MS accumulated more mutations compared to SS during the selective phase of the experiment (supplementary fig. S6, Supplementary Material online). This is consistent with a previous report showing that, for β-lactam antibiotics, the number of mutations accumulated in an adaptive laboratory experiment was higher in SS than in MS (Oz et al. 2014). Also, populations that evolved under MS need more generations to reach comparable resistance levels to SS and therefore MS promotes the accumulation of more mutations during its path towards β-lactam resistance. Moreover, in high antibiotic concentrations, serial dilutions are performed in populations with a lower yield (in our experiment, IC90), thus imposing a severe bottleneck that can lead to stochastic clearance of low-frequency mutants in the SS regime.

Supplementary tables S2–S3, Supplementary Material online and figure 1*E* show mutations that reached a frequency higher than 10% in at least one replicate population. For SS, we identified three mutations with a 100% frequency by the end of Phase 1: an IS1-mediated mutation in *acrR* [a transcriptional regulator of an efflux pump operon (Wang et al. 2001; Olliver et al. 2004; Pena-Miller et al. 2013) that modulates the level of expression of *acrAB* (Ma et al. 1996)], a non-synonymous mutation in *rpoD* (a RNA polymerase σ70 factor), and an intergenic IS-mediated short indel that involves clpX and *lon* genes that encode proteases which are known to share substrates (Smith et al. 1999). ClpX is part of the ClpXP two-component protease in *E. coli* that is involved in cell division through FtsZ degradation (Camberg et al. 2011) and therefore associated with resistance to antibiotics that target the cell wall (Bæk et al. 2014), while Lon is an ATP-dependent protease associated with a wide range of cellular activities (Luo et al. 2008), including regulating transcript levels of *marA* and their target genes *acrR*, *acrA*, and *tolC* (Nicoloff and Andersson 2013; Bhaskarla et al. 2016). Lon has been shown *in vitro* to modify drug-resistance levels when there is a loss of function (Matange 2020; Zou et al. 2021). Both proteins, ClpX and Lon, have been associated with increased resistance (Bæk et al. 2014; Liu et al. 2022).

MS mutations reaching 100% frequency by the end of Phase 1 were two non-synonymous substitutions affecting *ftsI*, a gene involved in cell division (Yao et al. 2012) that has been previously reported as a β-lactam-resistant determinant (Ghigo and Beckwith 2000; Buddelmeijer and Beckwith 2004; Oz et al. 2014; Batra et al. 2021), and a non-synonymous point mutation in *phoQ* (a large IS-mediated deletion of 40 genes). Also, two intergenic IS-mediated insertions involving *mgrB* and *yobH* reach a very high frequency (70%); *mgrB* is a small transmembrane protein produced in the PhoPQ signaling system, which has been shown to increase adaptation to stressful environments by remodeling the lipid A of the outer membrane (Barbosa et al. 2017, 2019; Kato et al. 1999; Prost et al. 2007).

In our experiment, only mutations in *clpX/lon* were found in replicates of both selection regimes. We also found that MS exhibited a certain degree of parallelism, with four mutations (including resistance mutations in *ftsI* and *dauA/prs*) present in more than one replicate population (red squares in fig. 1*E*). Conversely, none of the mutations identified in populations evolving under SS was present in more than one replicate, consistent with previous studies arguing that high genetic drift imposed by strong bottlenecks can reduce parallel evolution by increasing genetic variation across replicate populations (Mahrt et al. 2021).

### Rapid Resistance Acquisition is Unstable in Drug-free Environments

To further explore the mutational profile associated with different selection regimes, we sequenced 53 representative colonies isolated from the evolved populations. As anticipated by the population sequencing, mutations in *phoQ* and *ftsI* were prevalent in the MS regime. Other resistant mutations, notably *dauA/prs*, were found in both selection regimes (a complete list of mutations can be found in supplementary table S5, Supplementary Material online).

We cultivated in drug-free media these clonal populations and obtained time-resolved optical densities that we used to characterize the mutant’s growth dynamics. Growth rates of clones evolved under strong and mild selection regimes were significantly different (two-tailed t-test, p- value<0.05; supplementary fig. S3, Supplementary Material online), with isolates obtained from the mild-selection regime presenting, on average, an increased growth rate (mean=10.59, s.e.=0.332, n=29) in the absence of antibiotics with respect to clones that had evolved under SS (mean=9.69, s.e.=0.199, n=89).

Moreover, we used a flow cytometer to measure the relative frequency at the end of a competition assay of the ancestral and evolved populations against a fluorescent susceptible bacteria (Wiser and Lenski 2015) (see Methods for details). As illustrated in supplementary figure S4, Supplementary Material online, we were not able to observe significant differences in competitive fitness between populations that evolved under SS and MS. Interestingly, when populations were cultivated in drug-free media before the competition assay, populations previously evolved in SS completely compensated their fitness cost (mean=1.02, s.e.=0.01; t-test p- value<0.05), suggesting that mutations evolved under SS are unstable in the absence of selection.

**Fig. 2.**
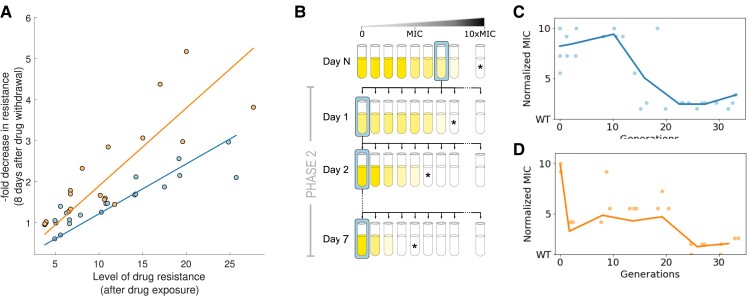
Evaluating the stability of resistance of populations evolved under different selection regimes. (*A*) Reduction in resistance after a period of relaxed selection (Phase 2) appears to be negatively correlated with the level of resistance achieved after the adaptive ramp (Phase 1). Each point corresponds to a population sampled at different time-points of Phase 1 (every time there was a measurable increase in MIC of the population). Lines denote best-fit linear regression for data obtained for each regime ($R^{2}=0.844$ and $R^{2}=0.714$ for MS and SS, respectively). Note how populations that evolved under SS appear to be more unstable than mutations emerging under mild selection, independently of the degree of resistance. (*B*) Schematic representation of Phase 2 of the evolutionary experiment consisting of transferring the evolved populations obtained at the end of Phase 1 into a drug-free environment, and performing a 7-day serial dilution protocol in the absence of selection for resistance. (*C*) Reduction in resistance measured daily during Phase 2. Dots represent a dose–response experiment for different replicate populations, and blue solid line is the mean over all replicates (N=4). (*D*) Populations evolved under SS present a rapid reduction in resistance after growing in a drug-free environment for eight serial dilutions (∼30 generations).

To evaluate the stability of resistance mechanisms acquired during the selective phase of the experiment, we sampled populations from different time points of Phase 1 (every time there was an increase in resistance) and performed serial dilutions in drug-free environments for seven days (∼30 generations). As shown in figure 2*A*, stability of resistance genes appears to be negatively correlated with the level of drug resistance they provide, independently of the selective regime ($R^{2}=0.844$ for MS, and $R^{2}=0.714$ for SS). For populations with comparable resistance levels, those that evolved under SS exhibited a reduction in resistance relative to their MS counterparts after counter-selection (slope of the best-fit line: 0.121 for MS and 0.189 for SS; ANCOVA p- value<0.05). This is consistent with previous studies showing that the higher the selective pressure, the more rapidly resistance is cleared from the population once the antibiotic is removed (Andersson and Hughes 2010; Barbosa et al. 2021; Card, Jordan, et al. 2021).

Following Phase 1, we transferred all replicate populations into drug-free media and performed serial dilutions in a constant environment (Phase 2 illustrated in fig. 2*B*). After seven days without selection for resistance, all replicate populations exhibited a significant reduction in MIC relative to the end of Phase 1 (two-tail t-test, p- value<0.05, df=6). In contrast, we found no significant differences in resistance levels after the drug-free period between selection regimes (two-tail t-test, p- value=0.2, with SS and MS reducing 89.5%±0.45 and 81.8%±9.6 their resistance levels, respectively). Crucially, despite presenting a similar reduction in resistance after counter-selection, both selection regimes exhibited different dynamics of resistance loss during Phase 2; MS maintained high levels of resistance levels for more than ∼10 generations, in contrast to SS, that reduced more than half its resistance during the first generations.

### Stable Resistance Mutations Identified through Genomic Analysis

After the 7-day period of relaxed selection, we sequenced three populations of each selection regime and found that resistance mutations in *phoQ* and *ftsI*, that were previously detected at 100% in MS during Phase 1, were still fixed in the population at the end Phase 2. Mutations in *dauA/prs* and *mgrB/yobH* were present at lower frequencies during Phase 1 and were observed in 100% of the population after removing selection for resistance (see supplementary tables S2 and S3, Supplementary Material online).

In contrast, mutations in *rpoD* were present at 100% during Phase 1 in SS but were completely cleared from the population during the selection-free period, probably due to a high cost previously reported for mutations affecting subunits of RNA polymerases (Jordan et al. 2022). Resistance mutations in *acrR* and *clpX/lon* identified in the strong selective regime in 100% of the population decreased in frequency and were observed at 60% after the non-selective phase. Also of interest is the presence of a non-synonymous mutation in the sensor histidine kinase *envZ*, observed at a 100% frequency by the end of Phase 2.

**Fig. 3.**
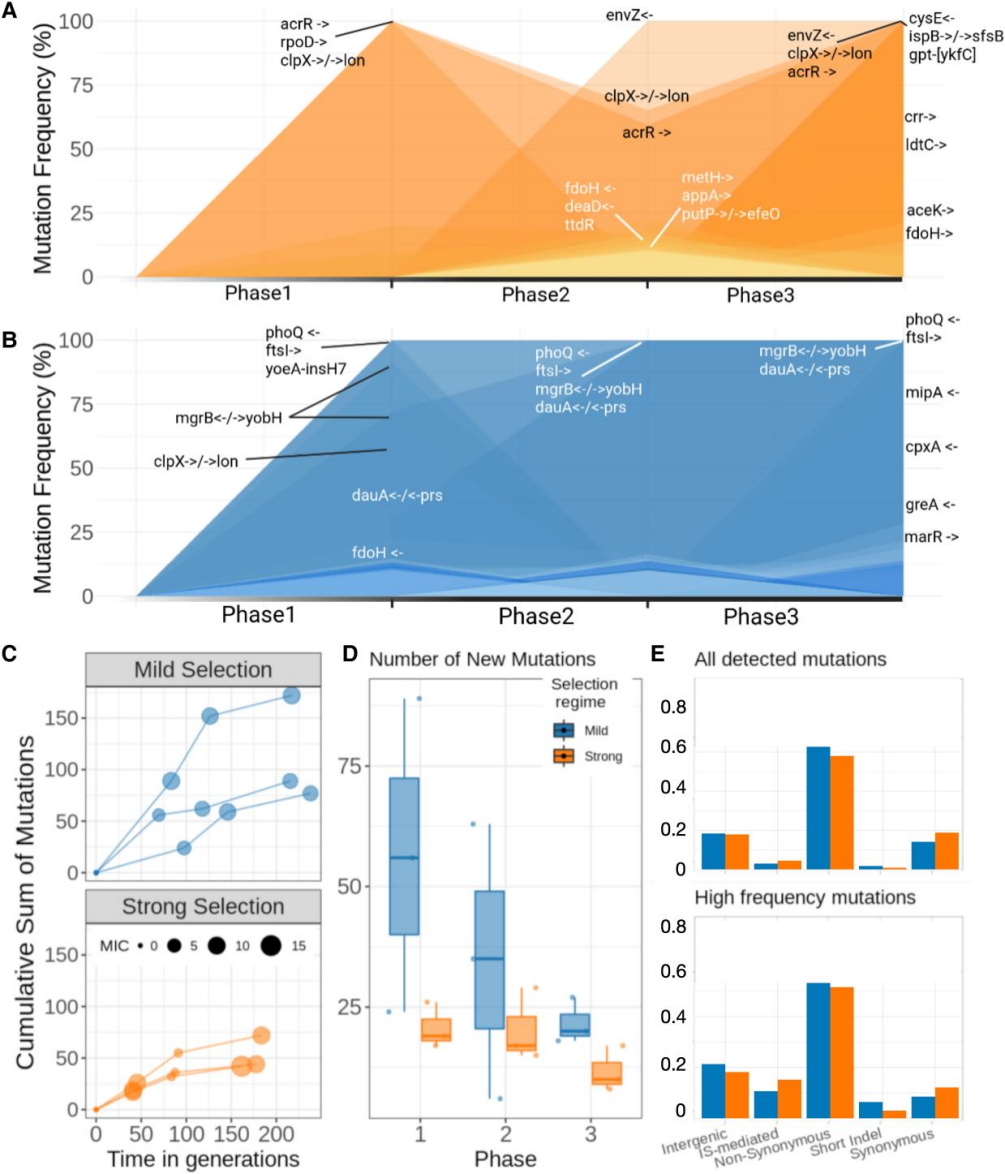
Genomic analysis of evolutionary experiment. (*A*,*B*) The trajectory of mutations with a frequency greater than 10% over the different phases of the experiment (top: SS, bottom: MS). Note how resistant mutations that reach 100% frequency during Phase 1 under SS (i.e. *acrR*, *clpX/lon*, and *rpoD*) are unstable in the absence of selection, thus reducing in frequency during Phase 2. In contrast, resistant mutations appearing at the end of Phase 1 under mild selection are stable in the absence of selection (notably, *phoQ* and *ftsI*), and can even continue to increase in the absence of the antibiotic (*dauA/prs* and *mgrB/yobH*), resulting in an accelerated rate of adaptation when antibiotics are re-introduced into the environment. (*C*) For each treatment, the accumulation of mutations per replicate in time (generations). Dot size represents the MIC level at the end of each phase. Note that Phase 1 is longer for MS than SS, it takes twice the number of generations for MS populations to reach the MIC. Phases 2 and 3 comprise a similar number of generations in both cases. (*D*) Number of new mutations in each phase. Only mutations not present in the previous phase are taken into account. Mild selection shows more variation between replicates. (*E*) Top panel: Proportion of mutations types for both treatments. All detected mutations are considered without filtering by frequency in the population. Bottom panel: Proportions of mutations types for both treatments, only mutations detected in more than 10% of the population in at least one phase are taken into account.

We also sequenced populations evolved under MS at the end of Phase 3 (see fig. 3*A*). Crucially, for the MS regime, resistance mutations in *phoQ*, *ftsI* and *dauA/prs* present in the population since Phase 1 can still be found in 100% of the population at the end of Phase 3. Other mutations, notably *dauA/prs* and *mgrB/yobH* that appeared during Phase 1 and increased in frequency during Phase 2, were observed at 100% at the end of Phase 3. Other mutations that rapidly increased in frequency when antibiotics were reintroduced were a short insertion in the *mipA* coding region, a small deletion in the coding region of *cpxA*, and a mutation in the multidrug efflux pump subunit *acrB* that appeared during Phase 3 and reached fixation.

For SS, mutations in *clpX/lon* and *acrR* that decreased in frequency during Phase 2 recovered and were found in 100% of the population at the end of Phase 3 (fig. 3*B*). Mutations in *rpoD* that were cleared during Phase 2 could not be found in the population after antibiotic reintroduction. A protein involved in the pathway peptidoglycan biosynthesis, LdtC, was observed at a high frequency by the end of Phase 3. We also sequenced clones, both from the evolved and the control populations, to confirm the presence of mutations observed in the sequenced populations (supplementary fig. S8 and table S5, Supplementary Material online).

As illustrated in figures 3*C* and 3*D*, MS populations accumulated more mutations than SS in all phases (supplementary fig. S6, Supplementary Material online). As expected for populations under selection, both regimes exhibited high non-synonymous to synonymous SNP ratios. Intergenic mutations were also similar in both regimes, while IS-mediated mutations were more frequent during SS. Interestingly, though IS-mediated mutations are a small proportion of all detected mutations, their proportion increases when we take into account only high-frequency mutations (above 10%; fig. 3*E*). As expected, IS-mediated mutations detected during Phase 1 appear to be reversible, in particular for MS where only a single IS-mediated mutation was maintained in the population in the absence of selection. For populations evolved under SS, IS-mediated mutations decreased in frequency during Phase 2 but were not cleared from the population (supplementary fig. S7, Supplementary Material online).

### Acceleration in Rate of Adaptation is Contingent on Evolutionary History

The genomic analysis showed that resistance mutations selected under different selection regimes have diverging stability profiles in the absence of selection. To probe if the loss of resistance observed in SS was associated with a fitness cost compensation occurred during the selection-free period, we performed competition experiments between samples obtained at the end of each phase against a susceptible strain and observed a significant increase in relative fitness after relaxed selection (two-tail t-test p- value<0.05) in contrast to MS (two-tail t-test p- value=0.27).

To evaluate if these diverging stability patterns in resistance mutations influence the rate of adaptation upon antibiotic reintroduction, we sampled populations from the end of Phase 2 and re-started an adaptive ramp following the same protocol as before. As in Phase 1, we observed a rapid increase in resistance in all populations during Phase 3, regardless of the treatment protocol used. Again, we continued the serial dilution experiment until all replicate populations reached at least a 10-fold increase in MIC (mean MIC=12.8±4.3 for MS, and mean MIC=13.4±11.8 for SS). Supplementary figure S5, Supplementary Material online shows the MICs of populations and clones sequenced throughout all experiment phases.

**Fig. 4.**
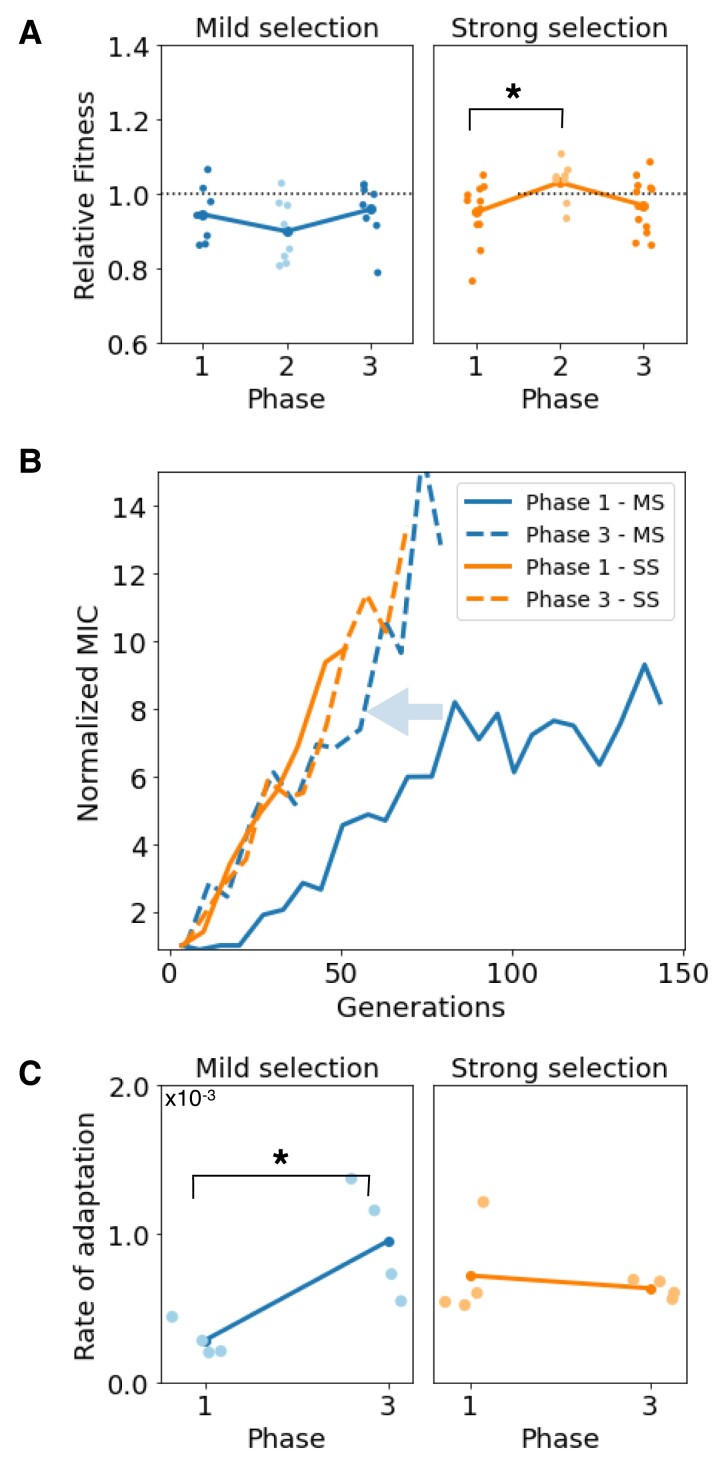
Rate of adaptation when antibiotics are reintroduced into the environment depends on previous drug exposures. (*A*) Fitness of populations sampled at the end of each phase relative to a fluorescent *E. coli*. During the non-selective phase of the experiment (Phase 2), populations evolved under SS completely compensated for the cost associated with resistance, conversely to populations evolved under MS that exhibited a higher fitness cost. In Phase 3, all populations exhibit a reduced fitness cost compared to the susceptible strain. (*B*) Increase in resistance as a function of time (measured in generations); solid lines represent the first adaptive ramp (Phase 1) and dotted lines the adaptive ramp inoculated with samples obtained from the end of Phase 2. Note how phenotypic trajectories are similar for SS (orange lines), contrarily to MS (blue lines), where resistance adaptation is significantly faster in Phase 3 compared with Phase 1 (acceleration highlighted with the arrow). (*C*) Comparison between rates of adaptation estimated for Phase 1 and Phase 3 (left: MS, right: SS) shows that resistance accelerated under MS, but not under SS.

In Phase 1, populations evolved under MS adapted significantly more slowly than those in the SS regime. In contrast, during Phase 3, adaptation to the antibiotic ramp followed similar trajectories in both selective regimes. As illustrated in figure 4*B*, a 10-fold increase in resistance was achieved after 49.9±11.9 generations and 44.8±6.4 generations for MS and SS, respectively. For MS, this is a significant increase in adaptation rate, as a 10-fold increase in of resistance was achieved until 106.2±38.4 generations during Phase 1. Also of note, MS achieved higher levels of resistance during Phase 3 compared to Phase 1.

We computed the rate of adaptation of both selection regimes under selection. As shown in figure 4*C*, for SS, we did not observe a significant change in the rate of adaptation between both selective phases (t-test p- value=0.35; $H_{0}$: mean rate of adaptation in both phases are equal). In contrast, during Phase 3, MS exhibited a higher rate of adaptation compared to Phase 1 (two-tailed t-test p- value<0.005), suggesting that evolutionary history can influence resistance adaptation to subsequent drug exposures.

### Population Dynamics Modeling of Resistance Adaptation in Dynamic Environments

**Fig. 5.**
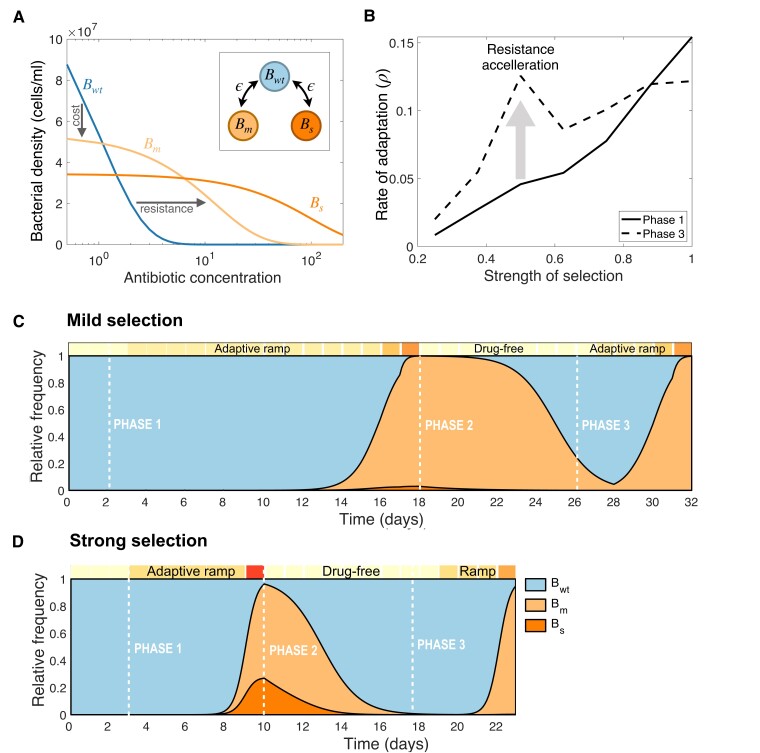
Numerical simulations of the population dynamics model. (*A*) Theoretical dose-response curves estimated for each bacterial strain: a susceptible wild-type ($B_{\mathrm{wt}}$), a mildly-resistant type ($B_{m}$) and a strongly resistant strain ($B_{s}$). Resistant mutations occur at a rate ϵ (see inset). A trade-off between resistance and fitness-cost is represented by resistant strains surviving at higher drug concentrations, albeit with a reduced density at lower doses with respect to $B_{\mathrm{wt}}$. (*B*) Rate of adaptation as a function of the strength of selection. Solid lines represent the rate of adaptation computed for Phase 1, while dotted lines denote the rate of adaptation estimated for Phase 3. The increase in rate of adaptation is maximized at intermediate strengths of selection; therefore, we argue resistance acquisition accelerates (gray arrow). (*C*,*D*) Relative frequencies of each bacterial type as a function of time computed by numerically simulating the evolutionary experiment; from an initial population composed exclusively of $B_{\mathrm{wt}}$ cells, we simulate a serial dilution experiment (boxes on top of each plot denote the environmental conditions: drug-free in light color and high drug concentrations in dark). After solving the system for two days in drug-free media, we simulate an adaptive ramp with different strengths of selection (Phase 1). (*C*) considering mild selection, and, (*D*) under strong selection. In both cases, once the level of resistance has achieved a 10-fold increase relative to the first day, the antibiotic is withdrawn from the environment for 7 days (Phase 2), before re-starting the adaptive ramp (Phase 3). Crucially, the duration of Phase 3 is shorter than Phase 1, suggesting that drug resistance adaptation accelerated at intermediate selective pressures.

To further explore the interaction between the strength of selection and the evolutionary history, we used a simple mathematical model consisting of multiple subpopulations exposed to a bactericidal antibiotic and competing for a single exhaustible resource. First, we assumed the population is composed of three bacterial types: a susceptible bacteria, denoted $B_{\mathrm{wt}}$, and two resistant subpopulations, $B_{m}$ and $B_{s}$, emerging from $B_{\mathrm{wt}}$ through a single-point mutation occurring at a rate ϵ (see inset diagram in fig. 5*A*).

Suppose we denote with S≥0 the concentration of a single limiting resource. In that case, we can model its uptake by a bacterial population through a function denoted u(S), a Monod-type term that depends on the extracellular resource concentration (Smith and Waltman 1995; Peña-Miller et al. 2012a). Therefore, growth rate of different strains can be characterized by the following growth kinetic parameters: ρ, denoting the resource conversion rate, K the half-saturation constant, and a maximum resource uptake rate, μ.

In our experimental system, we observed a trade-off between the degree of resistance and fitness cost in the absence of antibiotics, a property that we can express theoretically by considering that, at low antibiotic concentrations, $u_{\mathrm{wt}}(S)>u_{m}(S)>u_{s}(S)$. To model the bactericidal effect of AMP, we consider that the killing rate of each bacterial type is proportional to its density and the environmental drug concentration, with κ denoting the killing efficacy of the antibiotic. So, if $\kappa_{\mathrm{wt}}>\kappa_{m}>\kappa_{s}$ then, at high antibiotic concentrations, resistant strains (i.e. $B_{m}$ and $B_{s}$) present larger bacterial densities than the susceptible strain ($B_{s}$), as illustrated in figure 5*A*.

By numerically solving the model (equations described in the Methods and with parameter values defined in supplementary table S6, Supplementary Material online) we observed that, similarly to the experimental data, the rate of adaptation for the MS regime was significantly lower than for SS, with a 10-fold increase in resistance achieved in almost twice as many transfers for MS compared to SS. At the end of the adaptive ramp (Phase 1), the population structure was significantly different in both cases. In contrast to MS, where the population consisted almost entirely of $B_{m}$, the population evolved under SS exhibited the presence of both resistant types ($B_{m}$ and $B_{s}$). As a result, when removing the antibiotic from the environment (Phase 2), both drug-resistant subpopulations are outcompeted by the susceptible strain and cleared from the population. In our numerical experiments, the rate of decay was faster under SS (fig. 5*D*) than when simulating an MS adaptive ramp (fig. 5*C*).

Consequently, when antibiotics are reintroduced into the system after seven seasons of relaxed selection, a 10-fold increase in resistance is achieved faster than during Phase 1 for both selective regimes. For MS, resistance adaptation during Phase 3 was more than 60% faster than in Phase 1, while for SS, the same level of resistance was reached 25% faster. We repeated this numerical experiment for adaptive ramps with increasing selective pressures and found that, as anticipated by the experimental data, the rate of drug-resistance adaptation is maximized at intermediate strengths of selection (gray arrow in fig. 5*B*).

## Discussion

Antimicrobial substances are the main selective agents responsible for the evolution and dissemination of antibiotic resistance genes, both in the environment (Berendonk et al. 2015) and in clinical settings (Medeiros 1997). But evolutionary change is not only driven by the deterministic force of natural selection but also by genetic variation produced by random mutations. Moreover, mutations may exhibit epistatic interactions and express different phenotypes when present in different genetic backgrounds (Card et al. 2019; Lukačišinová et al. 2020; Rousset et al. 2021). The complex interaction between the strength of selection and contingencies imposed by pre-existing genetic variation can have profound consequences on microbial evolutionary dynamics, with examples ranging from the evolution of innovation (Meyer et al. 2012) and loss-of-function (Galardini et al. 2019), to compensation (Levin et al. 2000) and reversibility of costly mutations (Crill et al. 2000; Rebolleda-Gómez and Travisano 2019).

We used experimental evolution to probe the effect of different selective pressures and evolutionary histories in resistance adaptation during recurrent drug exposures. Similarly to previous studies (Jahn et al. 2017; Mahrt et al. 2021), during the selective phase of our experiment, the rate of adaptation was proportional to the strength of the selective pressure. Furthermore, we found that adaptive ramps with different strengths of selection resulted in populations with analogous fitness in environments containing high doses of AMP. Importantly, despite exhibiting similar phenotypes, the genomic analysis of the evolved and ancestral populations revealed that the intensity of the selective pressure was an important factor in the resulting genotypic profile (Oz et al. 2014; Mahrt et al. 2021). A consequence of exhibiting genetic heterogeneity is that when the environment changes, then the fitness cost associated with resistance mutations can also be highly variable. For instance, a previous study showed that strongly selected mutations enhanced cross-resistance to other antibiotics compared to mutants selected under MS (Oz et al. 2014).

In this manuscript, we focused on evaluating the stability of resistance mutations when selection is removed from the environment. We found that strongly selected mutants presented reduced fitness compared to the susceptible strain and were cleared from the population once the antibiotic was withdrawn from the environment. In particular, we found that drug-resistance mutations in acrR and *rpoD* selected in the SS regime were cleared during Phase 2. In contrast, resistance mutations in *ftsI* and *phoQ* evolved under MS were stably maintained in the population throughout all phases of the experiment. The stability of resistance mutations can be problematic when characterizing the level of resistance of clinical isolates. Indeed, our data suggest that equally resistant strains (analogous MIC, but with different mutational profiles resulting from diverging evolutionary histories) can modify the evolutionary trajectories towards β-lactam resistance in subsequent drug exposures. However, precisely because the strength of selection and evolutionary history interact to select and maintain genes in the population, it should not be expected that resistance genes identified in our *in vitro* experimental system would exhibit similar resistance and stability profiles in a clinical setting.

Another important aspect driving bacterial evolutionary dynamics is bottlenecks size (Vogwill et al. 2016; Garoff et al. 2020; Mahrt et al. 2021; Windels et al. 2021). In our experimental system, we considered a relative bottleneck size that is akin to scenarios of low- and high- selective pressures; in low-drug environments [e.g. drug-polluted natural environments (Martinez 2009)], the population size is large and the strength of selection is mild, while in high-drug environments (e.g. clinical use of antimicrobials) populations usually undergo severe bottlenecks (e.g., transmission between host, host’s immune system, tissue heterogeneity). To explore the role of different bottleneck sizes, we adapted our population dynamics model and assumed that a fixed initial density of cells is inoculated at the beginning of each season, with subpopulation frequencies obtained from the end of the previous season. Supplementary figure S10, Supplementary Material online illustrates that intermediate selection promotes the stability of resistant mutations in the population, resulting in an accelerated rate of adaptation upon antibiotic introduction. Note, however, that the increase in the rate of adaptation is less than when considering a relative bottleneck size. This is expected, as stronger bottlenecks are susceptible to stochastic effects and a greater rate of loss of beneficial mutations to genetic drift (Vogwill et al. 2016; Moxon and Kussell 2017).

The experimental protocol used in this study was designed to evaluate the evolutionary dynamics of bacterial populations in response to recurrent periods of selection, similar to those imposed by sequential treatment protocols. But environments that alternate selective pressures for and against resistance mutations can also be found elsewhere, particularly when considering that the health of humans, the environment, and animals are interconnected (Hernández-González and Castillo-Ramírez 2020). Indeed, drug-polluted natural environments are ubiquitous due to the use of antimicrobial substances in agriculture and as animal growth promotion (Szekeres et al. 2018; Zhou et al. 2018; Martinez 2009), with mounting evidence demonstrating a correlation between anthropogenic drug pollution and selection for antibiotic-resistance in human pathogens (Berendonk et al. 2015; Kraemer et al. 2019; Hernández-González and Castillo-Ramírez 2020). Altogether, our theoretical and experimental results suggest that sub-lethal doses of antibiotics found in natural environments, which, in principle, only impose a mild selection for resistance (Gullberg et al. 2011; Fuentes-Hernández et al. 2019), are sufficient to maintain highly resistant mutations at low frequencies in the population, thus enhancing the rate of resistance adaptation when exposed again to high doses of antibiotics.

## Materials and Methods

### Strains and Culture Condition

All experiments were conducted using *Escherichia coli* MG1655 grown in M9 minimal media supplemented with 1 g/l of casaminoacids, 2 g/l of glucose, and 5% of glycerol. All cultures were grown in 96-well plates with a volume of 200 μl, incubated at a temperature of $30^{\circ }C$ and shaken at 150 rpm using an orbital incubator. We used ampicillin sodium salt (Sigma A0166) in stock at 100 mg/ml diluted in water.

### Antibiotic Susceptibility Determination

To determine the susceptibility to the antibiotic, we performed dose–response curves to Ampicillin in 96 microtiter plates with a maximum volume of 200 μl. The experiment started with a stock solution of Ampicillin at a concentration of 100 mg/ml, which we diluted to make a dose–response curve consisting of 22 concentrations supplementary table S1, Supplementary Material online. Cultures in the plates were incubated at a temperature of $30^{\circ }C$, and shaken at 150 rpm using an orbital incubator. After 22 h of growth, optical density at 630 nm (OD) using a plate reader (BioTek EL808x). To estimate the MIC of the population, we used dose–response experiments in liquid media by exposing the population to a logarithmically spaced vector of drug concentrations. We estimated the percentage of inhibition based on the final OD of each population with respect to the control with no antibiotic. By numerically interpolating the dose–response curve, we estimated the critical doses used for MS (50% inhibition, IC50), and for SS (90% inhibition, IC90).

### Experimental Evolution

Our experimental protocol is adapted from Oz et al. (2014). For the evolutionary experiments, the antibiotics dilutions of all 96-well plates were prepared with an OpenTrons pipetting robot using a bespoke script coded in Python. The experiment is based on four replicates of a dose-response curve of Ampicillin with a range of 22 different concentrations (see supplementary table S1, Supplementary Material online). We evolved eight populations in parallel following two strategies: four populations were sampled each day based on from populations that grew on concentrations where inhibition achieved at least 90% (referred to as SS), and also populations that exhibited 50% inhibition (MS) with respect to the drug-free control. The volume of each selected populations was diluted at 1:5 in fresh medium, before transferring a sample of each population into a dose–response experiment (with the same range of drug concentrations as before). We repeated this serial dilution protocol until all populations achieved a 10-fold increase in resistance (8 days for SS, and 22 days for MS). Every time we observed an increase in the MIC in one of the replicates, we froze the population at $-80^{\circ }C$ for subsequent phenotypic and genotypic analysis.

We then continued the serial dilution protocol for seven additional days in the absence of antibiotics (Phase 2), and measured the MIC of the evolved populations every day using the same range of drug concentrations as before. Finally, Phase 3 consists in transferring the resulting populations to the adaptive ramp described in Phase 1. All the experiment was done following the previous protocol, the cultures were grown for 22 h, and we estimated the final optical density with a plate reader (BioTeK EL808x) and measured the MIC as before, these populations were kept in the freezer for subsequent analysis. To compute the generations elapsed during each transfer, we first convert the optical density measured each day (OD${}_{600}$, in arbitrary units) into cells per milliliter. This was achieved by estimating the number of cells per unit volume using a flow cytometer for cultures with a range of optical densities, and extrapolating from the best-fit line (slope=8.6048 and intercept=11.353). We then estimated the number of generations elapsed using the expression $\log_{2}\;(B_{i}/(\eta \cdot B_{i-1}))$, where $B_{i}$ denotes the number of cells per milliliter at the end of transfer i, and η>0 a constant dilution parameter (in this experiment η=0.02). Raw data and Python code used for analysis and visualization can be found in https://github.com/ccg-esb-lab/evoamp/.

### Genome Sequencing

Whole-genome sequencing was performed for populations and some clones at the end of the three phases of the experiment. For sampling clones, frozen samples from the evolutionary experiment from the three Phases were inoculated using 150 μl of each sample in four Petri dishes with LB agar using pearls at an appropriate dilution and selected all the individual clones that appeared on the plate after one day of incubation at $30^{\circ }C$; then we grew each clone in a 96-well plate in M9 with the appropriate glucose and incubated at $30^{\circ }C$ for 24 h with liquid media to obtain growth curves and enough culture to isolate DNA. The growth rate of each sample was analyzed using the *fitderiv* program (Swain et al. 2016). Using the growth rate, we performed a K-means algorithm to generate groups that have similar growth rates. The algorithm randomly selected one member from each group for sequencing. Genomic DNA was isolated from each clone using the DNeasy Blood & Tissue, QIAGEN kit.

For the population-level sequencing, frozen samples from evolutionary experiments were revived overnight and gradually increased the volume and concentration of Ampicillin until the populations reached a high optical density and survive in a concentration of 10× MIC (20 μg/ml). We first sampled 2 of the 4 replicas for each experiment at the end of the three phases, two for MS and two of SS; Genomic DNA was isolated from each population using the DNeasy Blood & Tissue, QIAGEN kit. We then sampled 2 more populations in Phase 1 and Phase 3, one of each treatment and we revived them overnight in LB media, without antibiotics, for four days for Phase 1 and six days for Phase 3. The populations were diluted 1:5 and gradually increased in volume and ampicillin concentration until populations reached a high optical density and survive in a concentration of 10 MIC (20 μg/ml). Genomic DNA was isolated from each population using the DNeasy Blood & Tissue Kit, QIAGEN.

All samples were genotyped by Illumina WGS using a NextSeq platform in a 2×75 bp paired-end configuration. We sequenced clones of each population with 40× coverage and populations with 100× coverage for each sample. Reads were trimmed using Trimmomatic (Bolger et al. 2014) with the following parameters: LEADING:3 TRAILING:3303 SLIDINGWINDOW:4:15 MINLEN:50. Clones and populations variant calling analysis was performed with breseq v0.35.7 (Deatherage and Barrick 2014), using default parameter settings. Option -p was used when analyzing population samples. We used *Escherichia coli* K12 substrain MG1655 genome as reference (Refseq sequence: $NC_{0}00913.3$). Comparisons between different phases of the experiment and with the reference genome were performed and were compared with gdtools from breseq. To assess the impact of mutations on antibiotic resistance we filtered out mutations that were also present in controls, and mutations that never reached a frequency of 10% in different phases of the experiment For the analysis of genotypes, we choose three replicas of the four of each treatment, with a control at the end of the experiment and the ancestral strain for each treatment, resulting in a total of 28 time points. Sequencing data were deposited at the NCBI under the BioProject ID PRJNA771356.

### Competition Experiments

Wild type *E. coli* MG1655 strain harboring the pFPV25.1 plasmid (*E. coli*+ pFPV25.1), which expresses the *gfpmut3a* gene from the rpsM constitutive promoter (Valdivia and Falkow 1996) was used to evaluate the potential growth penalty attributable to the mutations generated in evolved populations in competition experiments. *E. coli*+ pFPV25.1 strain and evolved populations (see supplementary table S2–S3, Supplementary Material online) were grown 14 h in 5 ml of LB Broth-Lennox (Condalab) supplemented or not with Ampicillin (2 mg/ml) at $30^{\circ }C$ with shaking (230 rpm). Then, these bacterial cultures were inoculated (1:100 in LB) and grown at $30^{\circ }C$ with shaking up to reach an OD at 630 nm of 0.6 (3.5–4 h), corresponding to an exponential growth phase. Next, mixed cultures (competitive growth) were started by inoculating pairs of the bacterial cultures (*E. coli*+ pFPV25.1 combined with any of the evolved populations) at a ratio of approximately 1:1 (250+250 μl) in 125-ml flasks containing 25 ml of fresh LB without antibiotics and incubated at $30^{\circ }C$ with shaking for 6 h. At 0 and 6 h, culture samples were taken to estimate the relative fraction of each of the two strains using flow cytometry. Briefly, samples containing ∼10^8^ cells were taken from bacterial mixed cultures and washed and resuspended in 1 ml of 0.22-μm-pore-size filtered 1X phosphate-buffered saline (PBS). Then, 50,000 events per sample using 525/40 nm filter (GFP) were read on a Beckman Coulter Life Sciences CytoFLEX flow cytometer. Quantitative measurements and distribution of fluorescence were determined with the CytExpert software program (Beckman Coulter Life Sciences).

### Population Dynamics Model

We used a simple mathematical model of microbial growth under resource limitation to study the evolutionary dynamics of a clonal bacterial population exposed to increasing concentrations of antibiotics. Bacterial growth rate was modeled as a saturating function of the environmental resource concentration, S, with the Monod term: u(S)=μB/(K+B) where μ represents the maximum resource uptake rate and K the half-saturation constant. Therefore, bacterial growth rate of each bacterial strain is given the resource uptake function multiplied by ρ, a resource conversion coefficient that represents the efficiency of each bacterial type in converting resource molecules into biomass.

For simplicity, we consider three bacterial types: a susceptible wild-type ($B_{\mathrm{wt}}$), a mildly-resistant strain ($B_{m}$), and a strongly-resistant strain ($B_{s}$). If we denote the concentration of antibiotic as A≥0, then bactericidal activity can be represented by parameter κ (by definition, $\kappa_{\mathrm{wt}}>\kappa_{m}>\kappa_{s}$). We consider that resistance is associated with a fitness cost in the absence of positive selection for genes encoding resistance mechanisms, by introducing constraints in parameter values such that, in low-drug environments, $u_{\mathrm{wt}}(S)>u_{m}(S)>u_{s}(S)$ (see supplementary table S6, Supplementary Material online for parameter values used). Resistance acquisition occurs by a single point mutation occurring at a rate 0<ϵ≪1. We consider that resistance can be reversed at a rate ϵ, but that $B_{s}$ cannot mutate into $B_{m}$, or vice versa.

Then the population dynamics occurring in a single day can be written as a set of differential equations:

(1a) $$\frac{dS}{dt}=-u_{\mathrm{wt}}(S)\cdot B_{\mathrm{wt}}-u_{m}(S)\cdot B_{m}-u_{s}(S)\cdot B_{s},$$

(1b) $$\frac{dA}{dt}=-A(\alpha_{\mathrm{wt}}B_{\mathrm{wt}}+\alpha_{m}B_{m}+\alpha_{s}B_{s}),$$

(1c) $$\begin{array}{rl} \frac{dB_{\mathrm{wt}}}{dt}= & (1-2\epsilon )\rho_{\mathrm{wt}}\cdot u_{\mathrm{wt}}(S)\cdot B_{\mathrm{wt}}+\epsilon (\rho_{s}\cdot u_{s}(S)\cdot B_{s}\\ & +\rho_{r}\cdot u_{r}(S)B_{r})-\kappa_{\mathrm{wt}}B_{\mathrm{wt}}A, \end{array}$$

(1d) $$\begin{array}{rl} \frac{dB_{m}}{dt}= & \;(1-\epsilon )\rho_{m}\cdot u_{m}(S)\cdot B_{m}+\epsilon \rho_{\mathrm{wt}}\cdot u_{\mathrm{wt}}(S)\cdot B_{\mathrm{wt}}\\ & -\kappa_{m}B_{s}A, \end{array}$$

(1e) $$\frac{dB_{s}}{dt}=(1-\epsilon )\rho \cdot u_{s}(S)\cdot B_{s}+\epsilon \rho_{\mathrm{wt}}\cdot u_{\mathrm{wt}}(S)\cdot B_{\mathrm{wt}}-\kappa_{s}B_{s}A.$$

Now, to model a serial dilution experiment we will consider that each transfer has a duration of T hours, with t∈[0,]T. Therefore, the state of the system in day i can be represented with the vector $x^{i}(t)=(S^{i}(t),A^{i}(t),B_{\mathrm{wt}}^{i}(t),B_{m}^{i}(t),B_{s}^{i}(t))$. In particular, to reflect that the evolutionary experiment considers an initial population consisting exclusively of susceptible cells, we consider that the initial conditions the first day are $x^{0}(0)=(S^{0}(0),A^{0}(0),B_{\mathrm{wt}}^{0},0,0)$. For subsequent days, i>0, a relative bottleneck can be modeled by considering that the initial conditions of the system can be written as $x^{i}(0)=(S^{i}(0),A^{i}(0),B_{\mathrm{wt}}^{i}(0),B_{m}^{i}(0),B_{s}^{i}(0))=(S_{0},A_{0}^{i},\eta \cdot B_{\mathrm{wt}}^{i-1}(T),\eta \cdot B_{m}^{i-1}(T),\eta \cdot B_{s}^{i-1}(T))$, where η>0 represents a dilution parameter, and $S_{0}$ is a fixed parameter denoting the daily initial concentration of limiting resource. To model absolute bottlenecks, we assume the total initial density to be fixed (*B*_0_ = 1 × 10^6^), with a population structure determined from the frequencies of each strain at the end of the previous transfer. Finally, the drug concentration used each day, $A_{0}^{i}$, is defined based on the treatment regime under consideration. Numerical simulations of the model were performed in Matlab, with scripts available in https://github.com/ccg-esb-lab/evoamp/.

## Supplementary Material

msac185_Supplementary_Data

## Data Availability

The data and code underlying this article are available in GitHub at https://doi.org/10.5281/zenodo.7080457.
