# Supplementary material for: Evolutionary History and Strength of Selection Determine the Rate of Antibiotic Resistance Adaptation
Source: Mol Biol Evol. 2022 Sep 5;39(9):msac185. doi: 10.1093/molbev/msac185 (PMC9512152; doi:10.1093/molbev/msac185)
Supplement: msac185_Supplementary_Data [file msac185_supplementary_data.zip › EvoAMP_MBE_supp.pdf]

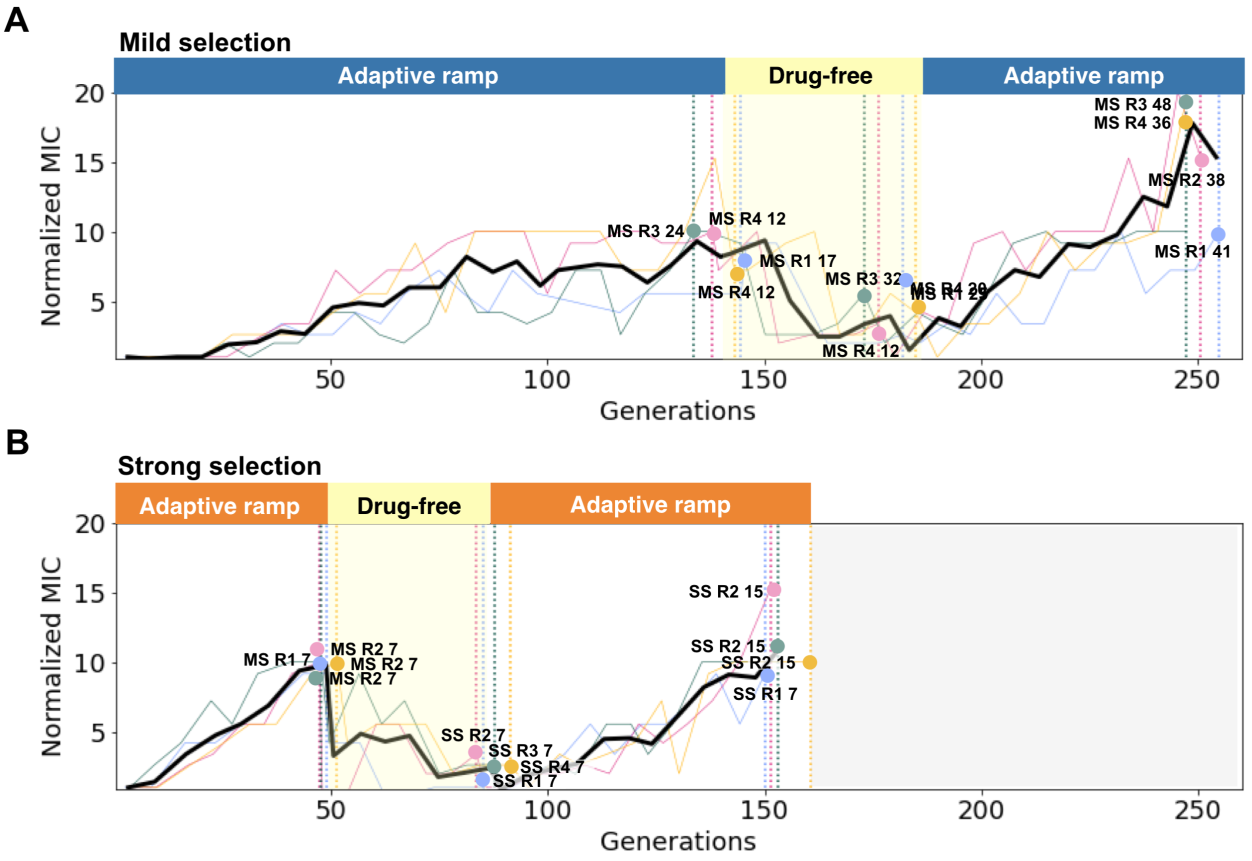

Figure S1. Normalized resistance during all phases of the evolutionary experiment. Black line denote the mean resistance and colored lines represent each replicate population ($N = 4$). Circles denote replicate populations that were sampled for genome sequencing. A) Mild selection. B) Strong selection.

MS	Phase 1																							
	1	2	3	4	5	6	7	8	9	10	11	12	13	14	15	16	17	18	19	20	21	22	23	24
R1	1	1	2	1	2	4	4	2	4	4	6.8	2	8.4	6.8	8.4	6.8	8.4	5.2	4	6.8	1	1	2	1
R2	1	1	2	1	2	5.2	5.2	6.8	5.2	5.2	4	4	8.4	8.4	11.2	6.8	5.2	1	1	8.4	1	4	5.2	4
R3	1	1	2	1	2	2	2	4	4	8.4	2	2	5.2	5.2	5.2	4	2	4	2	8.4	1	1	6.8	2
R4	1	2	2	2	4	4	4	4	5.2	6.8	6.8	4	8.4	11.2	14.4	11.2	6.8	6.8	2	8.4	2	14.4	6.8	6.8

MS	Phase 2						
	1	2	3	4	5	6	7
R1							
R2							
R3							
R4							

MS	Phase 3															
	1	2	3	4	5	6	7	8	9	10	11	12	13	14	15	16
R1	2	4	2	1	1	5.2	8.4	5.2	1	4	1	1	11.2	1	4	2
R2	2	4	2	2	6.8	6.8	8.4	8.4	18.4	8.4	14.4	11.2	14.4	18.4	6.8	8.4
R3	2	2	2	1	5.2	6.8	8.4	6.8	1	6.8	11.2	14.4	14.4	8.4	14.4	14.4
R4	2	2	4	5.2	6.8	8.4	11.2	11.2	18.4	30.8	20	20	14.4	20	20	30.8

SS	Phase 1						
	1	2	3	4	5	6	7
R1	2	2	5.2	8.4	6.8	14.4	11.2
R2	2	2	5.2	6.8	5.2	11.2	8.4
R3	2	5.2	6.8	8.4	8.4	18.4	20
R4	2	2	5.2	8.4	11.2	11.2	18.4

SS	Phase 2						
	1	2	3	4	5	6	7
R1							
R2							
R3							
R4							

SS	Phase 3															
	1	2	3	4	5	6	7	8	9	10	11	12	13	14	15	16
R1	1	2	4	6.8	6.8	8.4	14.4	11.2	14.4	18.4	11.2	18.4	11.2	14.4	30.8	20
R2	2	2	5.2	4	5.2	5.2	11.2	8.4	14.4	14.4	11.2	20	20	20		
R3	2	4	6.8	5.2	11.2	14.4	6.8	11.2	20	20	14.4	30.8	11.2	30.8	30.8	30.8
R4	4	6.8	5.2	6.8	8.4	14.4	6.8	18.4	20	20	30.8	20	18.4	30.8	30.8	30.8

Figure S2. Daily AMP concentration of the population that is selected for the serial transfer. Each row represents a different replicate, top for MS and bottom for SS. The color of each box corresponds to the drug concentration (in $\mu g/ml$) from which the population was transferred to the following season (white for no drug and dark red for 30 times the MIC). During PHASE 1 and PHASE 3, the drug concentration used for the serial transfer increases constantly as the population becomes resistant to the antibiotic. Between both adaptive ramps, we implement a drug-free period of seven days (PHASE 2).

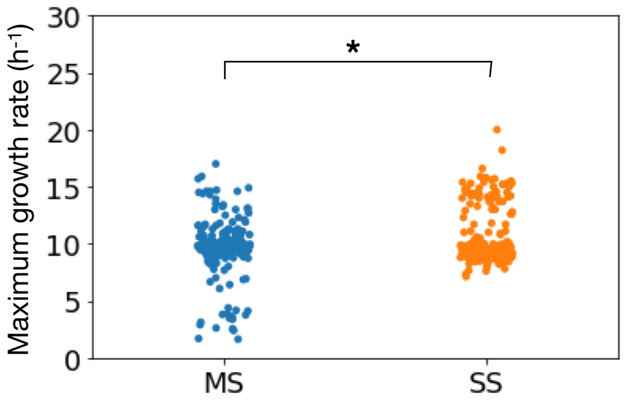

Figure S3. Maximum growth rate estimated for sequenced clones from growth kinetic curves. Note that clones evolved under MS (in blue) present increased growth rate with respect to clones that evolved under SS (in orange; two-tailed t-test, p-value< 0.05).

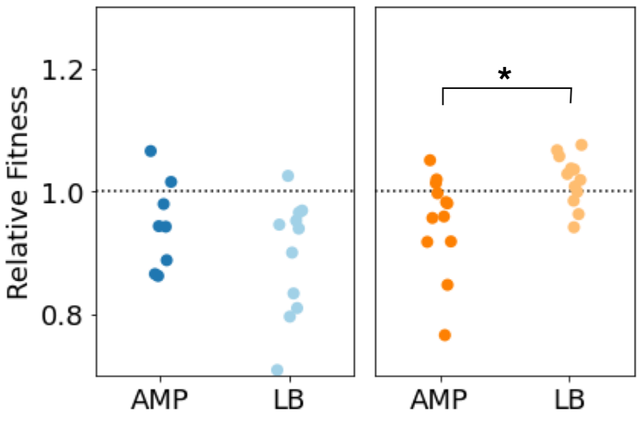

Figure S4. Relative fitness of populations sampled at the end of PHASE 1 compared to a susceptible strain. Note that both resistant populations have reduced fitness compared when grown in selective media, but after only two days in drug-free media the cost of the SS mutant has been completely compensated, in contrast to MS that presents a stable fitness cost.

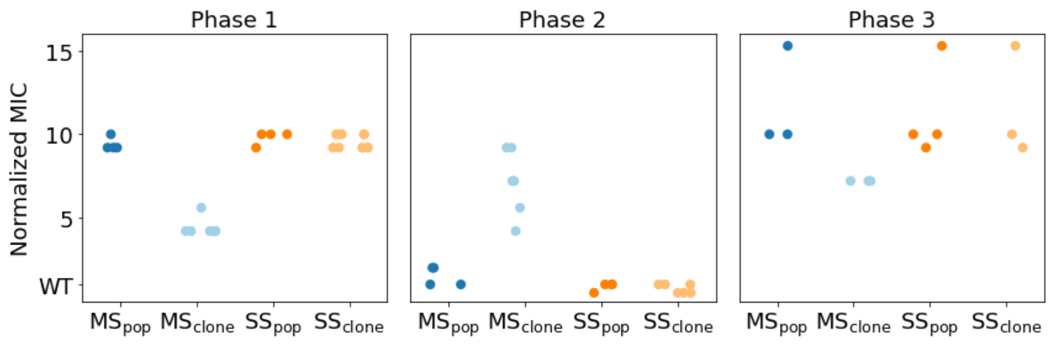

Figure S5. MIC estimated for each evolved population at the end of each phase. Each dot represents a sequenced population/clone (dark colors for populations, light colors for clones; blue for MS and orange for SS).

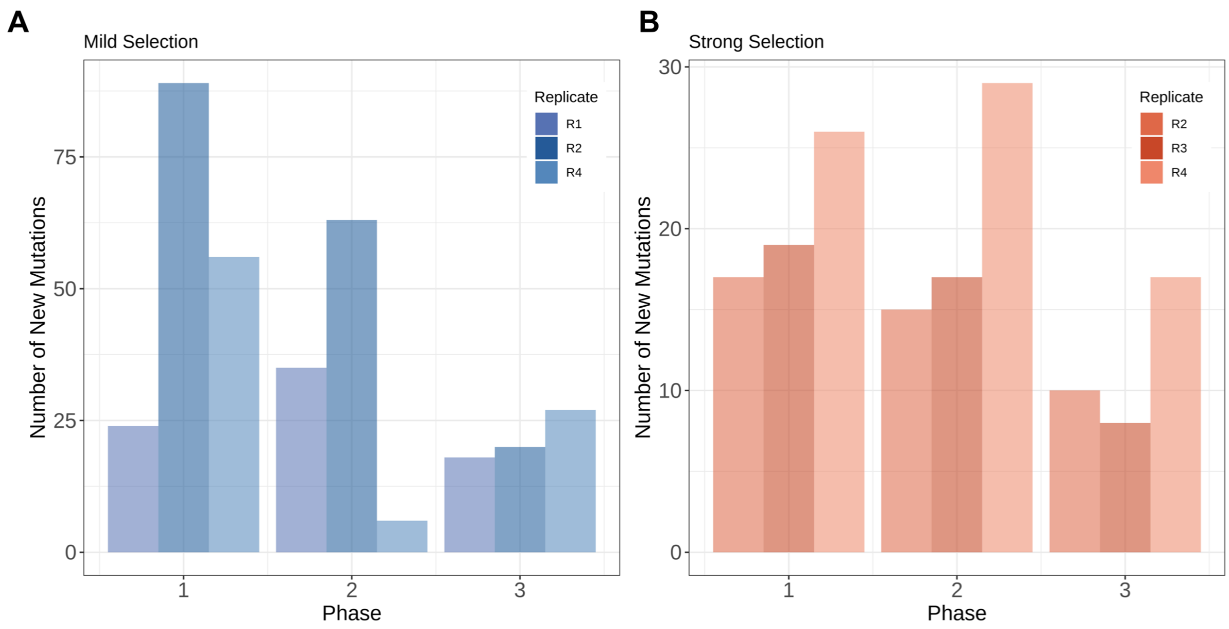

Figure S6. Number of new mutations that appear in each population replicate. A) New mutations in each replica at the end of each phase for the populations evolved under mild selection. B) New mutations in each replicate at the end of each phase for the populations evolved under SS. Also, the number of new mutations is higher on average in replicates evolved under MS than in SS.

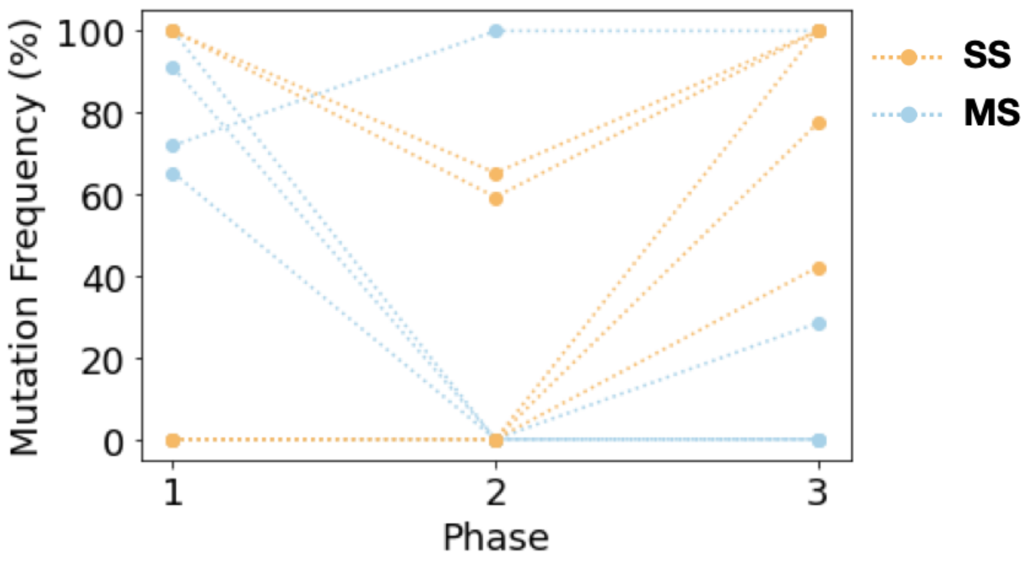

Figure S7. Frequency of IS-mediated mutations during the evolutionary experiment. In orange, mutants evolved under SS, and in blue under MS. As expected, during the non-selective phase of the experiment (PHASE 2), most IS-mediated mutations decreased in frequency in the population.

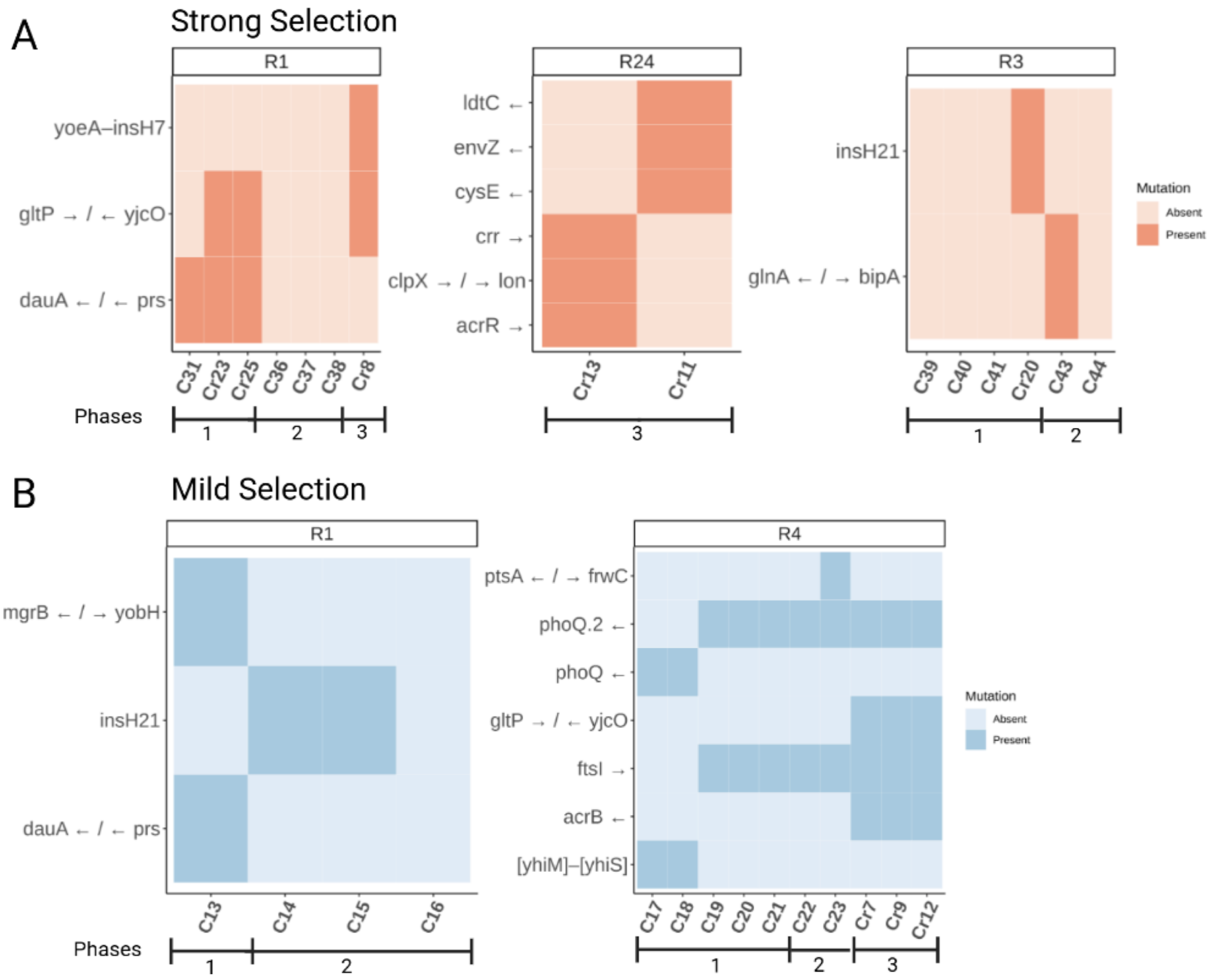

Figure S8. **Mutations found in individual clones selected from different replicates of both treatments.** A) Strong selection, B) Mild selection. Most mutations detected at high frequencies in the populations were also found in different clones. One big exception is the case of a nonsynounymous mutation in *rpoD* found in the SS regime replicate 2, during PHASE 1 only, for which we do not have sequenced clones. For Mild Selection mutations observed in replicates 1 and 4 were confirmed. There are no sequenced clones for replicate 2.

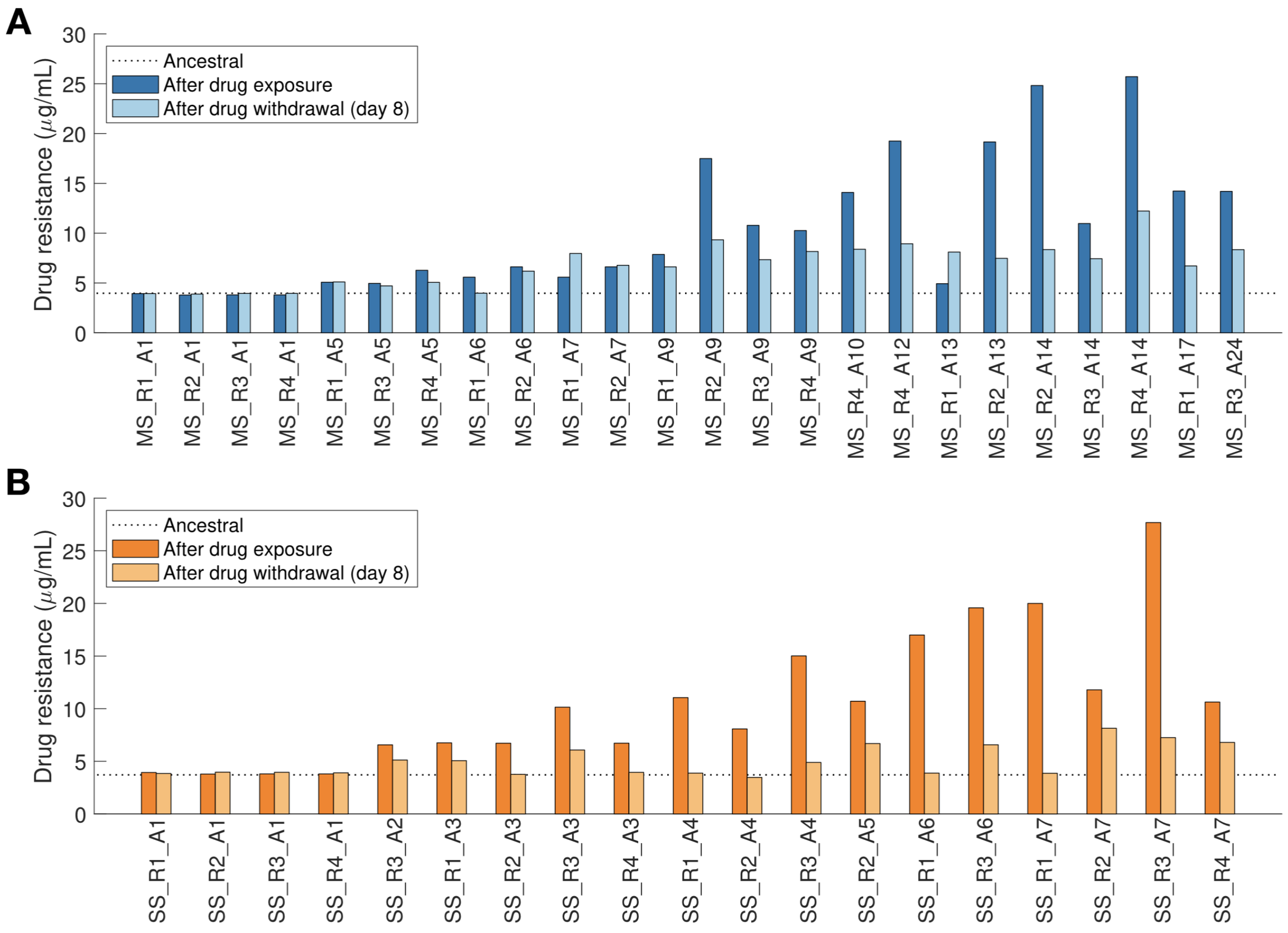

Figure S9. **Stability of resistance is associated with the level of resistance.** Bar plots illustrating drug resistance levels estimated for sample populations obtained during the adaptive ramp (dark color), and after seven days of relaxed selection (light color). Replicate populations were selected from PHASE 1 and every time there was an observable increase in resistance. A) Mild selection (blue). B) Strong selection (orange).

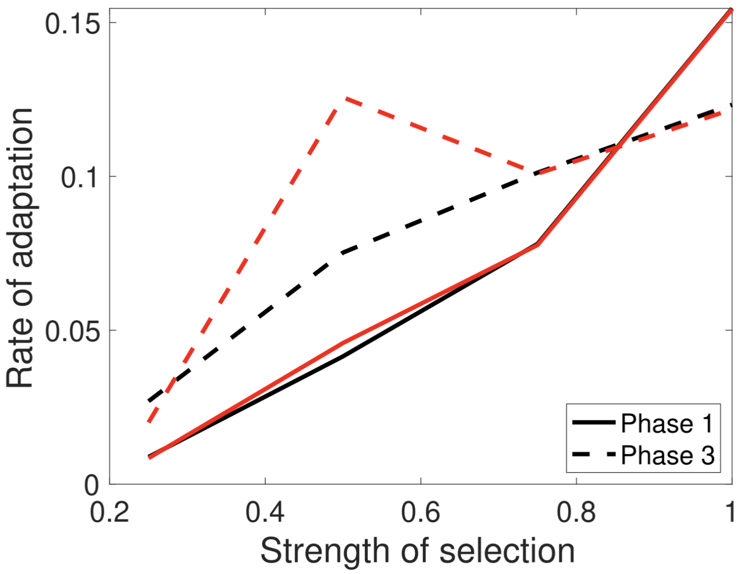

Figure S10. **Interaction between strength of selection and rate of adaptation under different bottleneck scenarios.** Rate of adaptation for adaptive ramps with different strengths of selection simulated using the population dynamics model. Solid lines illustrate that, during PHASE 1, the rate is a monotonously increasing function of the strength of selection. The dotted lines denote the rate of adaptation achieved in PHASE 3 of the experiment (red using a relative bottleneck and black using an absolute bottleneck). In both cases, the rate of adaptation accelerated at intermediate selective strengths, although more dramatically when considering relative bottlenecks.

Table S1. Ampicillin concentrations used in the dose-response experiments.

Key	Concentration (μg/ml)	MIC relative to WT
M	0	0
D1	1	0.5
D2	2	1
D3	4	2
D4	5.2	2.6
D5	6.8	3.4
D6	8.4	4.2
D7	11.2	5.6
D8	14.4	7.2
D9	18.4	9.4
D10	20	10
D11	30.8	15.4
D12	40	20
D13	52	26
D14	68	34
D15	86	43
D16	111.2	55.6
D17	144	72
D18	184	92
D19	240	160
D20	309.6	154.8
D21	400	200

Table S6. Parameters used in the numerical solutions of the population dynamics model

Parameter	Value	Description
ρ_*	$(1.5\times10^9, 1.5\times10^9, 1.5\times10^9)$ cell μg^{-1}	resource conversion coefficient (B_{wt},B_m, B_s)
μ_*	$(3.6\times10^{-9}, 2.25\times10^{-9}, 1.65\times10^{-9})\mu$g cell^{-1}h^{-1}	maximum uptake rate (B_{wt},B_m, B_s)
K_*	$(1,1,1)\mu$g ml^{-1}	half-saturation constant (B_{wt},B_m, B_s)
κ_*	$(1, 0.08, 0.01)$	antibiotic killing efficacy (B_{wt},B_m, B_s)
ε	1×10^{-8} per locus, per cell, per division	mutation rate
η	0.1% of biomass	dilution parameter
S_0	1μg ml^{-1}	resource supply concentration
T	24 hours	duration of each season

Table S2. List of mutated genes from the sequenced populations from the mild selection regime.

	Gene	Phase 1 (%)	Phase 2 (%)	Phase 3 (%)	Diversity	Category
Mild Selection	*clpX* → / → *Ion*	65.3	0	0	Indel	O/O
	dauA ← / ← *prs*	26.9	100	100	Intergenic SNP	P/F,E
	mgrB ← / →*yobH*	91.1	0	0	Indel	
	mgrB ← / →*yobH*	71.9	100	100	Indel	
	fadJ←	0	11	0	Non-Syn SNP	I
	ygfB ←	0	13.6	0	Non-Syn SNP	S
	panF →	0	0	12.4	Non-Syn SNP	H
	rpsE →	0	10.2	0	Syn SNP	J
	ftsI ←	100	100	100	Non-Syn SNP	M
	acnB →	0	10	0	Non-Syn SNP	
	paoD ←	10.4	11.3	11.1	Non-Syn SNP	O
	lacZ ←	10.3	0	8.1	Non-Syn SNP	
	acrB ←	0	0	100	Non-Syn SNP	M,P
	ybhR ←	0	13.9	0	Non-Syn SNP	M,P
	ycbF →	12.3	10.8	10.9	Non-Syn SNP	O
	appA →	0	14.4	0	Non-Syn SNP	M
	putP → / → *efeO*	0	16.4	0	Intergenic SNP	E/P
	putP → / → *efeO*	0	8.7	10	Intergenic SNP	E/P
	dauA ← / ← *prs*	0	100	100	Intergenic SNP	P/F,E
	ychS →	0	15.1	0	Non-Syn SNP	S
	ychS →	0	0	10.3	Non-Syn SNP	S
	rsxC →	13.2	9.7	12.2	Syn SNP	K,M
	rsxC →	0	11.4	15	Non-Syn SNP	K,M
	mipA ←	0	0	84.1	Indel	M
	fliF →	11.6	8.5	0	Non-Syn SNP	N
	yoeA–insH7	100	0	0	Indel	
	gatY ← / ← *fbaB*	0	0	13.9	Intergenic SNP	G
	gatY ← / ← *fbaB*	9.7	12.6	10.1	Intergenic SNP	G
	kgtP ← / → *yfiS*	11.8	0	0	Intergenic SNP	G/S
	thyA ←	13.3	0	0	Non-Syn SNP	L
	endA →	10.8	0	0	Non-Syn SNP	
	qseC →	0	10	0	Non-Syn SNP	N
	rnpB ←	10.6	10.7	8.5	Intergenic SNP	
	fdoH ←	22.6	16.9	0	Non-Syn SNP	G
	fdoH ←	12.9	0	0	Syn SNP	G
	yjcF ← / ← *actP*	9.9	11.2	8.7	Intergenic SNP	S/P
	yjfM →	14.7	0	0	Syn SNP	K
	lgoD →	0	10.9	0	Non-Syn SNP	
	ftsl →	100	100	100	Non-Syn SNP	M
	dnaX →	10.1	0	0	Non-Syn SNP	L
	phoQ →	100	100	100	Non-Syn SNP	T
	marR →	0	0	21.9	Indel	K
	cpxA ←	0	0	81.7	Indel	T
	fliF →	0	0	10.3	Non-Syn SNP	N,U
	greA ←	0	0	28.5	Indel	K
	aceK ←	0	0	18.8	Non-Syn SNP	T
	yjcF ← / ← *actP*	0	0	13.6	Intergenic SNP	S/R

Table S3. List of mutated genes from the sequenced populations from the strong selection regime.

	Gene	PHASE 1 (%)	PHASE 2 (%)	PHASE 3 (%)	Diversity	Category
Strong Selection	*yafD* →	0	10.4	0	Non-Syn SNP	S
	clpX → / → *Ion*	100	65.2	100	Indel	O/O
	acrR →	100	59.3	100	Indel	S
	crr →	0	0	77.5	Indel	G
	ttdR ←	0	17.6	0	Non-Syn SNP	K
	gpt-[ykfC]	0	0	100	Indel	
	proY →/→ *malZ*	0	13.9	8.4	Intergenic SNP	E/G
	ispB →/→ *sfsB*	0	0	100	Indel	H/K
	paoD ←	8.6	8.9	10.3	Non-Syn SNP	O
	fepA ←	0	10	0	Non-Syn SNP	P
	ybfA → / → *rhsC*	0	10.3	0	Intergenic SNP	M/S
	appA →	9.6	16.9	0	Syn SNP	
	putP → / → *efeO*	0	17.4	0	Intergenic SNP	E/P
	ldtC ←	0	0	42.2	Indel	M
	rsxC →	0	10.4	0	Syn SNP	K/M
	rsxC →	0	14.2	9.3	Syn SNP	K/M
	rsxC →	0	14.6	0	Non-Syn SNP	K/M
	wcaM ←	0	11	0	Non-Syn SNP	
	gatY ← / ← *fbaB*	0	0	13.6	Intergenic SNP	G
	yehL →	0	10.6	0	Syn SNP	
	yfcS ← / ← *yfcV*	0	12.4	0	Intergenic SNP	O
	rodZ ←	0	13.7	8.6	Non-Syn SNP	M
	kgtP ← / → *yfiS*	0	10.6	0	Intergenic SNP	G/S
	ttdR ←	0	21.1	0	Non-Syn SNP	K
	rpoD →	100	0	0	Non-Syn SNP	K
	deaD ←	0	22.8	0	Non-Syn SNP	J
	rpsE ←	0	10.2	0	Non-Syn SNP	J
	envZ ←	0	100	100	Non-Syn SNP	M
	cysE ←	0	0	100	Non-Syn SNP	
	xanP →	0	12.2	0	Non-Syn SNP	M
	fdoH ←	19.7	17.2	20.7	Non-Syn SNP	G
	aceK →	0	0	21	Non-Syn SNP	T
	metH →	0	17	0	Non-Syn SNP	J

Table S4. Functional categories

E	Amino Acid metabolism and transport
G	Carbohydrate metabolism and transport
H	Coenzyme metabolism
I	Lipid metabolism
J	Translation
K	Transcription
L	Replication and repair
M	Cell wall/membrane/envelop biogenesis
N	Cell motility
O	Post-trasnlational modification, protein turnover, chaperone functions
P	Inorganic ion transport and metabolism
S	Function Unknown

Table S5. Complete list of mutated genes from the sequenced clones.

	Replicate ID	Clone ID	Gene	Cluster	%	r_{max}	MIC
Mild selection	MS_R1_A17+M0	C2	*dauA* $\rightarrow$ / $\leftarrow$ *prs*, *mgrB* $\leftarrow$ / $\rightarrow$ *yobH*	2	100	6.86	3.4
	MS_R1_A17+M7	C50	*insH21*	1	94	9.48	2.0
		C9	*insH21*	2	2	9.48	2.0
	MS_R4_A12+M0	C9	*phoQ* $\leftarrow$, *[yhiM]–[yhiS]*	1	32.1	9.48	5.56
		C26	*phoQ* $\leftarrow$	2	7.1	7.77	15.0
		C16	*phoQ* $\leftarrow$, *ftsI* $\rightarrow$	3	3.6	13.0	7.2
		C8	*phoQ* $\leftarrow$, *ftsI* $\rightarrow$	4	53.6	10.5	7.2
		C10	*phoQ* $\leftarrow$, *ftsI* $\rightarrow$	6	3.6	17.0	7.2
	MS_R4_A12+M7	C5	*phoQ* $\leftarrow$, *ftsI* $\rightarrow$	1	77.1	9.47	9.2
		C13	*phoQ* $\leftarrow$, *ftsI* $\rightarrow$, *ptsA* $\leftarrow$ / $\rightarrow$ *frwC*	5	22.9	10.4	9.2
	MS_R4_A12+M7_RE16	C5	*phoQ* $\leftarrow$, *ftsI* $\rightarrow$, *acrB* $\leftarrow$, *gltP* $\rightarrow$ / $\leftarrow$ *yjcO*				
		C1	*phoQ* $\leftarrow$, *ftsI* $\rightarrow$, *acrB* $\leftarrow$, *gltP* $\rightarrow$ / $\leftarrow$ *yjcO*				
		C11	*phoQ* $\leftarrow$, *ftsI* $\rightarrow$, *acrB* $\leftarrow$, *gltP* $\rightarrow$ / $\leftarrow$ *yjcO*				
Strong selection	SS_R1_A7+M0	C6	*dauA* $\leftarrow$ / $\leftarrow$ *prs*	3	83.3	12.8	2.6
		C6	*dauA* $\leftarrow$ / $\leftarrow$ *prs*, *gltP* $\rightarrow$ / $\leftarrow$ *yjcO*				
		C2	*dauA* $\leftarrow$ / $\leftarrow$ *prs*, *glyP* $\rightarrow$ / $\leftarrow$ *yjcO*				
	SS_R1_A7+M7	C19		3	83.3	12.8	2.6
		C8		5	8.3	12.3	2.6
		C7		6	4.2	16.6	2.6
	SS_R1_A7+M7_RE16	C4	*yoeA–insH7*, *gltP* $\rightarrow$ / $\leftarrow$ *yjcO*				
	SS_R3_A7+M0	C80	*insH21*				
		C73		1	83.8	9.46	7.2
		C26		2	12.5	7.77	9.2
		C70		3	2.5	14	9.2
		C71		5	1.25	11.7	4.2
	SS_R3_A7+M7	C7	*typA* $\leftarrow$ / $\rightarrow$ *glnA*	1	74.4	16.6	4.2
		C38		2	20.5	8.32	9.2
	SS_R4_A7+M7_RE16	C5	*ldtC* $\leftarrow$, *envZ* $\leftarrow$, *cysE* $\leftarrow$				
